# Maternal Preeclampsia and Neonatal Outcomes

**DOI:** 10.1155/2011/214365

**Published:** 2011-04-04

**Authors:** Carl H. Backes, Kara Markham, Pamela Moorehead, Leandro Cordero, Craig A. Nankervis, Peter J. Giannone

**Affiliations:** ^1^Department of Pediatrics, College of Medicine, The Ohio State University and Nationwide Children's Hospital, 700 Children's Drive, Columbus, OH 43205, USA; ^2^Department of Obstetrics and Gynecology, College of Medicine, The Ohio State University, Columbus, OH 43210, USA

## Abstract

Preeclampsia is a multiorgan, heterogeneous disorder of pregnancy associated with significant maternal and neonatal morbidity and mortality. Optimal strategies in the care of the women with preeclampsia have not been fully elucidated, leaving physicians with incomplete data to guide their clinical decision making. Because preeclampsia is a progressive disorder, in some circumstances, delivery is needed to halt the progression to the benefit of the mother and fetus. However, the need for premature delivery has adverse effects on important neonatal outcomes not limited to the most premature infants. Late-preterm infants account for approximately two thirds of all preterm deliveries and are at significant risk for morbidity and mortality. Reviewed is the current literature in the diagnosis and obstetrical management of preeclampsia, the outcomes of late-preterm infants, and potential strategies to optimize fetal outcomes in pregnancies complicated by preeclampsia.

## 1. Introduction

Preeclampsia is a multisystem, highly variable disorder unique to pregnancy and a leading cause of maternal and fetal/neonatal morbidity and mortality [1–7]. While preeclampsia complicates 6%–10% of all pregnancies in the United States, the incidence is believed to be even higher in underdeveloped countries [8, 9]. Recent evidence suggests that preeclampsia accounts for approximately 15.9% of all maternal deaths in the United States and is a major cause of perinatal morbidity and death [10, 11]. Therefore, physicians must carefully weigh the risks to both mother and fetus in management decisions. To that end, optimal treatment strategies have not been fully defined, leaving physicians with incomplete data to guide their patient care practices [8, 12, 13]. The increased incidence of perinatal morbidity and mortality seen in pregnancies complicated by preeclampsia, although complex and multifactorial, is primarily due to the need for premature delivery and uteroplacental insufficiency resulting in a compromise of blood flow to the fetus [14, 15]. This paper will review the diagnosis and obstetrical management of preeclampsia, the outcomes of late-preterm infants, and potential strategies to optimize fetal outcomes in pregnancies complicated by preeclampsia.

## 2. Diagnosis of Preeclampsia

Antepartum diagnosis of mild, moderate, and severe preeclampsia is based on series of defined criteria occurring after 20 weeks of gestation [16]. Severe PE is defined as a blood pressure greater than 160 mm Hg (systolic) or 110 mm Hg (diastolic) associated with proteinuria greater than or equal to 5 grams per day. Furthermore, PE is regarded as severe in the presence of multiorgan involvement including thrombocytopenia (platelet count less than 100,000/uL), pulmonary edema, or oliguria (less than 500 mL per day). In contrast, mild PE is characterized by an elevated blood pressure less than 160 mm Hg (systolic) or 120 mm Hg (diastolic) with proteinuria greater than 300 mg, but less than 5 g, per day [9]. Ongoing debate on the optimal way to classify disease severity in preeclampsia is likely due to incomplete knowledge of the underlying pathophysiology of disorder, with the clinical and laboratory manifestations of preeclampsia representing a common endpoint for a variety of maternal disease states during pregnancy [17]. 

## 3. Current Obstetrical Practice

The only definitive cure for preeclampsia is delivery of the fetus and placenta [18]. Given the progressive nature of the disorder, delivery is often necessary to minimize maternal morbidity and mortality. On the other hand, one of the primary goals of obstetricians is to deliver infants who are functionally mature and capable of adapting to the extrauterine environment without the need for intensive care [19]. Therefore, in pregnancies complicated by preeclampsia, obstetricians must balance the need for achieving *in utero* fetal maturation with the maternal and fetal risks of continuing pregnancy, including progression to eclampsia, abruptio placentae, and HELLP syndrome, as well as fetal growth restriction and demise [17, 20–22]. At the present time, delivery is typically recommended for women who develop preeclampsia, regardless of disease severity, at 37 gestational weeks [23]. At this gestational age, the maternal and fetal risks during expectant management clearly outweigh potential benefits to the fetus. In addition, delivery is recommended for all women with severe preeclampsia no later than 34 weeks gestation [9, 24]. However, there are no clear guidelines addressing the optimal timing for delivery in women with mild preeclampsia between 34 and 36 weeks gestation (late-preterm gestation) who remain in stable condition [25]. Although is it believed that the majority of late-preterm deliveries in pregnancies with mild preeclampsia are due to maternal or fetal conditions warranting intervention, recent evidence suggests that iatrogenic elective late-preterm delivery remains a part of obstetrical practice [26]. For instance, a recent study of 1,850 women with stable mild gestational hypertension showed that over one-quarter of patients (25.5%), without any maternal or fetal indications, had iatrogenic elective late-preterm deliveries [27]. 

## 4. Consequences of Premature Delivery: Late-Preterm Infant Outcomes

Late-preterm births represent the fastest growing subset of premature births. Data from 1992 to 2002 show that two thirds of the decade's increase in preterm births was due to a rise in the incidence of late-preterm deliveries [28]. This increase was largely attributed to obstetrical and pediatric disciplines considering late-preterm infants functionally full term [29]. However, accumulating evidence suggests that late-preterm infants are, in fact, physiologically immature compared with infants born at term and are at significant risk for a broad range of complications [30–34]. 

### 4.1. Survival

Neonatal and infant mortality rates are consistently higher in late-preterm infants than in term infants [35–37]. A population-based study from the British Columbia Perinatal Database Registry investigated mortality statistics for a cohort of late-preterm infants (33–36 weeks, *n* = 6391) compared to term infants (37–40 weeks, *n* = 88, 867) from 1999–2002. The authors found that neonatal mortality (deaths among infants 0–27 days' chronological age) and infant mortality (death among infants 0–364 days' chronological age) was 5.5 and 3.5 times greater in the late-preterm group, respectively. Interestingly, the authors also noted that even after excluding the neonatal period, the risk of mortality between 28 days and 1 year was still 2 times higher in the late-preterm group [38]. This finding is consistent with previous epidemiological data from Canada and the United States showing an increase in the gestation-specific neonatal mortality rate of late-preterm infants between 6 and 8.5 times when compared with term infants [39, 40]. A study by Young and colleagues determined the relative risk for mortality rate for each weekly estimated gestational age cohort from 34–42 weeks, using 40 weeks as the reference cohort. The authors showed that mortality and the relative risk of death decreases with each increasing week in gestational age. Specifically, the infant mortality rates in pregnancies delivered at 34, 35, and 36 weeks gestation were 12.5, 8.7, and 6.3 times higher, respectively, compared to term (40 weeks) controls [41].

### 4.2. Morbidity

In the past, epidemiology and health service research, as well as patient-care guidelines, suggested that 34 weeks gestation was a surrogate for fetal maturity [42]. Infants born between 34 and 36 weeks gestation were labeled as “near-term” infants and were believed to be at a low risk for significant morbidities [43, 44]. This led to a relative lack of attention of the neonatal consequences when delivery was being considered beyond 34 weeks gestation [45]. However, a growing body of literature indicates that late-preterm infants are at greater risk for a number of complications, primarily respiratory problems. Several studies have shown that late-preterm infants are at increased risk for respiratory distress syndrome (RDS), transient tachypnea of the newborn (TTN), persistent pulmonary hypertension (PPHN), and respiratory failure compared to term infants [30, 46–49]. Evidence suggests that late-preterm infants have a nine times greater incidence of respiratory distress syndrome than term infants (28.9% versus 4.2%, *P* < .001) [34]. Importantly, evidence suggests a significant reduction in neonatal respiratory morbidity when gestation is extended beyond 34 weeks, a benefit seen for each week increase in gestational age up to term [50, 51]. In a population-based study of neonatal morbidity in the United States, the incidence of RDS was 7.4% at 34 weeks, 4.5% at 35 weeks, 2.3% at 36 weeks, and 1.2% at 37 weeks [50]. A more recent study noted infants born at 34 weeks were 18 times more likely to require supplemental oxygen for at least one hour and over 19 times more likely to require assisted ventilation compared to infants born at 38–40 weeks gestation [52]. This suggests that there is a significant portion of late-preterm infants with respiratory disorders and respiratory failure requiring intervention. 

## 5. Effects of Preeclampsia on Late-Preterm Infant Outcomes

### 5.1. Risk of Fetal Demise/Stillbirth

Stillbirth represents an important cause of fetal loss in the late-preterm infant [53]. Although greater than 90% of fetal deaths occur in the first 20 weeks of gestation, the rate of stillbirth is approximately 3 per 1000 live births beyond 28 weeks gestation. Interestingly, evidence suggests that beginning at approximately 36 weeks, the risk of intrauterine fetal demise increases substantially [54]. Severe preeclampsia represents significant risk factor for intrauterine fetal demise, with estimated stillbirth rate of 21 per 1000 [55]. In the setting of severe preeclampsia, as previously discussed, the risk of fetal death outweighs the potential benefits of pregnancy prolongation. However, in cases of mild preeclampsia, the risk of fetal demise is over 50% less than pregnancies with severe preeclampsia (stillbirth rate of 9 per 1000) [55]. Despite a paucity of data to guide clinical decision making in pregnancies with mild preeclampsia, obstetricians are left to balance the small, but important risks of fetal demise, with the benefits of pregnancy prolongation and potential for continued *in utero* maturation, particularly in pregnancies less than 37 weeks gestation.

### 5.2. Intrauterine Growth Restriction (IUGR)

Fetal growth is a useful marker for fetal well-being [56, 57]. Pregnancies complicated by intrauterine growth restriction (IUGR), defined as a pathological process of reduced fetal growth, have been associated with an increase in perinatal mortality [58, 59]. Preeclampsia, a condition characterized by decreased uteroplacental blood flow and ischemia, is a significant risk factor in the development of IUGR and represents the most common cause of IUGR in the nonanomalous infant. Data has consistently shown that for any given gestational age at birth, including term, a weight below the 10th percentile significantly increases the risk of mortality [60]. To that end, an infant at 38–40 weeks with a weight of 1,250 grams has a significantly greater mortality risk than one born of similar weight at 32 weeks [61]. It is important to note that while reduced birth size is associated with severe preeclampsia, it has not been as well described in pregnancies complicated by mild preeclampsia [62]. Ødegård et al.showed pregnancies complicated by severe preelampsia had infant birth weights 12% lower than expected, while pregnancies with mild preeclampsia showed no difference in weight gain from expected norms [63]. Previous guidelines have suggested that the late-preterm IUGR fetus should be delivered if there is any evidence of maternal hypertension [61]. However, the high risk of complication related to preterm delivery in the late-preterm infant, as well as the apparent negligible effect of mild preeclampsia on fetal growth and maternal health, highlight the importance of carefully selecting the appropriate time of delivery in pregnancies complicated by IUGR [64]. 

### 5.3. Hematologic Effects

Maternal preeclampsia can result in neonatal thrombocytopenia, typically defined as a platelet count less than 150,000/uL [65]. In pregnancies complicated by preeclampsia, thrombocytopenia is generally identified at birth or within the first 2–3 days following delivery, with resolution by 10 days of life in most cases [66]. Severity of thrombocytopenia related to preeclampsia is highly variable, with a small percentage of infants developing severe or clinically significant thrombocytopenia (<50,000/uL) [67, 68].The pathogenesis of thrombocytopenia among infants born to mothers with preeclampsia is unknown [69]. One potential mechanisms is that preeclampsia, and the resultant fetal hypoxia, has a direct depressant effect on megakaryocyte proliferation [70]. This is supported by studies showing that growth-restricted neonates have significant megakaryocytopoeitic defects without evidence of increased platelet destruction [71]. At present, there is a paucity of evidence-based recommendations to guide clinicians on which platelet counts warrant intervention [72]. Given the inherent risks of platelet transfusions, including the induction of a systemic inflammatory response and worsening of lung function immediately following the transfusion, additional studies are needed to guide clinical management [73, 74].

In addition to the well-described effects of preeclampsia on platelets, neonates delivered to women with preeclampsia have a 50% incidence of neutropenia (defined as absolute neutrophil count less than 500) [75]. Neutropenia has a variable course, typically lasting days to weeks in affected infants. The biological mechanism for preeclampsia resulting in neonatal neutropenia has not been fully elucidated. One potential mechanism is that preeclampsia, and the resultant uteroplacental insufficiency, inhibits fetal bone marrow production of the myeloid lineage manifested by a decrease in neutrophil production [66]. Neutropenia associated with maternal preeclampsia is also associated with reduced numbers of circulating colony forming unit-granulocyte macrophage (CFU-GM) and decreased neutrophil storage pools [76]. Neutropenia is generally self-limited although in some cases it may be severe enough to warrant therapy with granulocyte-colony stimulating factor (G-CSF) [77]. Although some studies suggest that there is an increased risk for nosocomial infection among affected neonates even after resolution of their neutropenia, other studies have demonstrated no increased propensity for infection [75, 78]. 

### 5.4. Bronchopulmonary Dysplasia (BPD)

Although the pathophysiology of preeclampsia is poorly defined, evidence suggests that abnormal placentation, characterized by shallow invasion of the maternal arteries, compromises uterine blood flow at the expense of the growing placenta and fetus [79]. The resulting hypoxia and ischemia may restrict fetal angiogenesis [80]. Considering the growing evidence suggesting that preservation of in utero vascular growth is critical in maintenance of alveolarization (“vascular hypothesis of BPD”), it is possible that preeclampsia may alter critical lung-vessel interactions necessary for normal lung development [81, 82]. A recent study shows that maternal preeclampsia is, in fact, associated with an increased risk for development of BPD, even after adjusting for gestational age, birth weight, and other clinical confounders (OR 2.96, 95% CI 1.17-7.51, *P* = .01) [83]. Additional studies have shown that BPD occurs in infants of mothers with preeclampsia, but only in the setting where preeclampsia is severe enough to lead to fetal growth restriction [82]. Ongoing research is needed to better understand the biological mechanisms linking *in utero* disruption of angiogenesis, including preeclampsia, and resultant impairments in fetal growth on important neonatal outcomes [84]. 

### 5.5. Neurodevelopmental Outcome

Given that preeclampsia is a heterogeneous disorder, it is not surprising that the neurodevelopmental outcomes of exposed infants are highly variable [85, 86]. Some evidence suggests that preeclampsia is associated with a decreased risk of cerebral palsy. These authors found a protective effect of maternal preeclampsia on cerebral palsy regardless of exposure to magnesium sulfate [87]. In addition, there is some evidence to suggest a lower incidence of IVH among infants born 26–30 weeks exposed to maternal preeclampsia compared to age-matched controls (4.8% versus 20.5%, *P* < .001) [86]. However, some data suggest infants born to mothers with preeclampsia have lower MDI scores (Bayley II scales of infant development) at 24 months of age compared to infants without maternal preeclampsia (*P* = .04) [88]. The association between maternal preeclampsia and worse neurodevelopmental outcomes has been challenged by more recent evidence suggesting that infants exposed to preeclampsia have, in fact, higher scores on developmental testing at 18 months corrected age [89]. Again, these differences highlight the fact that preeclampsia represents a common endpoint for a number of adverse maternal conditions and that efforts to better characterize subtypes of preeclampsia may allow for a clearer understanding of the impact of preeclampsia on short and long-term neonatal outcomes.

### 5.6. Fetal Origins of Adult Disease States

*In utero* development is characterized by rapid cellular and molecular growth. The ontological processes critical for maturation of the fetus are highly sensitive to alterations in the intrauterine environment [90]. Ongoing evidence suggests that various adult disease states (hypertension, obesity, diabetes) may begin during fetal development, and the insults from preeclampsia exposure accrued during sensitive periods of development may predispose an individual to an increased risk of disease in adulthood [91]. For example, a population-based study of over one million children exposed to preeclampsia showed an increased risk of endocrine, nutritional, and metabolic derangements during adolescence and early-adulthood (up to 27 years of followup) among the exposed cohort. These risk factors remain even after adjusting for differences in lifestyle (smoking, exercise, socioeconomic status, and diet) [92]. Epidemiological studies show that infants exposed to preeclampsia during gestation are associated with an increased risk of diabetes and cardiovascular morbidity in adulthood [93]. These studies underscore the concept that the physiologically immature fetus is highly susceptible to disruptions *in utero* placental blood flow and that insults from preeclampsia exposure accrued during critical periods of fetal development may predispose an individual to an increased risk of disease beyond the immediate postnatal period. Additional studies are needed to understand the causal pathways that may drive disordered fetal development in preeclampsia, as well as the potential impact of preeclampsia in altering expression of key genes involved in fetal programming and adult disease processes [94].

## 6. Medical Management: Optimizing Fetal Outcomes

There are a limited number of therapeutic options in the management of preeclampsia with known benefit to the fetus. Antepartum management routinely involves administration of antenatal steroids in anticipation of preterm delivery. Antenatal administration of corticosteroids for as few as 12–24 hours before delivery has been shown to decrease morbidity and improve survival rates of infants born before 34 weeks' gestation [95]. However, considering that the safety and efficacy of antenatal corticosteroids in the late-preterm infant remains unproven, additional studies are warranted [96]. Magnesium sulfate, a commonly used medication for seizure prophylaxis in women with preeclampsia, has been shown to have a neuroprotective effect on the preterm infant. A recent meta-analysis of over 6000 infants showed that antenatal magnesium sulfate given to women at risk for preterm birth decreased the incidence of cerebral palsy (RR 0.68, 95% CI 0.54–0.87) and gross motor dysfunction (RR 0.61, 95% CI 0.44–0.85) [97]. Prospective, randomized controlled trials have shown that magnesium therapy is associated with a decreased incidence of cerebral palsy among survivors exposed to the medication between 24 and 31 weeks gestation [98]. 

## 7. Conclusion

Historically, there has been a relative lack of consideration to the complications of premature delivery at greater than 34 weeks gestation, with the belief that 34 weeks is a surrogate marker for fetal maturity. Recent evidence suggests that infants born between 34 and 36 weeks gestation are, in fact, physiologically immature compared to term infants. Furthermore, given the potential for preeclampsia to disrupt mechanisms regulating fetal growth and development, a better understanding of the pathophysiology of the disorder may allow us to develop strategies to prevent morbidities from fetal through adult life. Because of the high variability of each case, a general recommendation for the optimal timing of delivery is not possible. However, based on the review of data, we believe that a multidisciplinary, collaborative approach between the fields of maternal-fetal medicine and neonatology is necessary to weigh the maternal and fetal risks of prolonging the pregnancy versus the potential benefits of further fetal maturation across most gestational ages.
